# Biomarkers and brains: situating dementia in the laboratory and in the memory clinic

**DOI:** 10.1080/14636778.2019.1652804

**Published:** 2019-08-13

**Authors:** Joanna Latimer, Alexandra Hillman

**Affiliations:** a Science & Technology Studies Unit, Sociology, University of York, York, UK; b School of Social Sciences, Cardiff University, Cardiff, UK

**Keywords:** animal models, biogerontology, mindedness, dementia, memory clinics, epigenetics

## Abstract

This paper provides a comparison of how genetic biomarkers are used (or not) in three contexts: clinic-based diagnostic work with people; lab-based research on mice and their marbles; and lab-based research on thrashing nematodes. For all the worldwide drive to find biomarkers that can be used in the detection of early, presymptomatic dementia, there is little research on how or when the association between biomarkers and a definitive disease are being made to “hold.” First, we show the disjuncture between the animal modeling that underpins laboratory attempts to stabilize genetic biomarkers and the paradigms that inform clinical diagnosis. Secondly, we develop this theme to show how in our third site, an epigenetics “worm” laboratory, neurodegenerative changes are explored as located in specific gene-environment interactions over time. We speculate whether such an enactment brings us closer to a notion of “situated biology,” to undercut possibilities of making genetic biomarkers of preclinical dementia hold.

## Introduction

There is an ongoing challenge for biomedicine to firm up biomarkers that can be used in the early detection of dementia (King 2018). These are best described as genetic biomarkers which can be used to identify those people at greatest risk of becoming demented in the future. Contemporary policy dictates that meeting this challenge is paramount if the quest for the early detection, risk prediction and prevention of dementias are to prosper. Yet problems arise when dementia science seeks to establish biomarkers that can be used presymptomatically. This is not just because they may result in misidentifying people as at risk (Milne *et al*. 2018), but also that they rely on a relatively stable association between the biomarkers and a discrete disease. In contrast, a post-mortem examination has shown that even where the “soma” displays biomarkers of Alzheimer's disease and other dementias (ADAD), there may be no clinical history of dementia (Leibing 2016). The reasons for why these discrepancies occur are complex and pertain to how dementia etiology and classification is being conceived and researched.

By “stabilizing dementia” we are addressing the extent to which the category “dementia” can be “made to hold” (Latour 1987) as an association between genetic biomarkers and a discrete, identifiable disease. The point is that this stability is required in order that the biomarkers can be used in the diagnosis of dementia prior to its clinical expression. Our concern is whether this stability can only be achieved by excluding contextual issues of great importance. Specifically, the growing critique around dementia science suggests that what is made absent from research that seeks to stabilize dementia biomarkers, especially those that can be used pre-symptomatically, is the minded, embedded and embodied human subject. For example, social scientists such as Lock (2014), Moser (2011), and Williams, Katz, and Martin (2011) question how a neuroscientific culture is somaticizing dementia: that is, making mind a matter of body, specifically the brain, in ways that relocate thinking, consciousness and even subjectivity as a matter of “cognition” and neurobiological substance (Hillman and Latimer 2019; Moser 2011).

Human mindedness can be understood not simply in terms of cognition, or brain matter, but in terms of consciousness and conscience, including the capacity to reason, think, feel, will, perceive, judge, sense and make sense. As such, mindedness is understood to be irreducible to neural substance or activity (the brain) (Gabriel 2017). Cromby (2015) puts his finger on what is at stake here:

At stake is the so-called “hard problem” of how physical processes in the body and brain can give rise to conscious experiences .... A collaborative analysis between a neuroscientist and a philosopher (Bennett and Hacker 2003) shows how in neuroscience this problem is frequently managed (in the sense of being concealed or bypassed, rather than solved) by simply treating the mind as though it were equivalent to the brain. (13)

As Cromby goes on to emphasize, Bennet and Hacker argue that these processes are mistaken since these processes can only logically be attributed to persons, not to “isolated parts.”

Moreover, social studies of dementia science increasingly also press for biological research aimed at identifying and stabilizing dementia biomarkers to give greater recognition of the subject whose mindedness is relational, located and contingent. Here, Lock (2014) has spoken of the need for a “local biology” that recognizes the contingent nature of how a body becomes visible *as* losing its mind as much, much more than a matter of how genes mutate or not. Niewöhner (2011) extending Lock’s notion of local biology, explores the significance of how epigenetics gets beyond the material basis of bodies and diseases as genetically determined, to reimagine the material basis of bodies as an effect of gene–environment interactions. Noting, however, that the laboratory enacts the environment as natural forces, Niewöhner argues for biology to take account of how bodies are embedded in socio-political, as well as natural environments, and presses attention to “biography” and “milieu” in the production of the material basis of bodies. In a more recent paper, Niewöhner and Lock (2018), drawing on Haraway’s (1988) notion of situated knowledges, press for research that can produce “situated biologies,” especially how “different forms of ‘local’ arise in environment/human entanglements,” and “how material agency becomes situated and contingent through various knowledge practices” (681).

We enter this debate by exploring how different locations enact dementia – particularly across the divide between human and non-human animals. Specifically, we compare and contrast how biomarkers are used across three contexts in which dementia is being enacted: clinic-based diagnostic work with people, lab-based research on mice and their marbles; and lab-based research on thrashing nematodes. Reflecting upon the alignments and tensions between these three enactments is especially important in the context of drives for biomarkers that can be used to stabilize dementia presymptomatically (Prince, Bryce, and Ferri 2011). This is because of the complexity of how research that stabilizes biomarkers does or does not take account of the situated and minded subject. In this respect, we attend closely to what is made absent by some practices and yet present by others (Woolgar and Lezaun 2013) in attempts to make dementia hold.

## In pursuit of biomarkers

The dominant hypothesis in dementia science determines ADAD as neurodegenerative, progressive pathologies that make themselves known over time, and that follows a linear trajectory. This theorization suggests that by the time ADAD makes itself present as clinical symptoms it is already too late to do anything about it (Aronowitz 2009). However, while a clinical diagnosis may be too late to reverse the disease, it may be possible to ameliorate its effects, since dementia emerges in the dementia science literature as more of a multiple (Mol 2002) than this hypothesis suggests. For example, there are (at least) two possible theories here: a notion that ADAD’s are different disease entities, along a spectrum perhaps, but with their own taxonomies; or of a “dementia continuum,” a syndrome (Fox *et al.* 2013), that is a “dimensional (lying along a continuum)” rather than a “categorical (representing a distinct entity)” construct (Walter 2010, 534). In addition, for some, the etiology that has come to predominate dementia science is the amyloid or “pathological cascade” hypothesis (Karran, Mercken, and De Strooper 2011) that also indicates ADAD happens over time and starts years before someone can be said to have dementia; while for others, dementia remains deeply correlated with growing old and with, for example, cardio-vascular change, that can be prevented through lifestyle activities (Leibing 2019; Livingston *et al*. 2017). Both constructions of dementia in their different ways have the possibility of shifting dementia from being a problem of old age to a problem of ageing, one that can be known and managed and even eventually treated (Perry, Zhu, and Smith 2013).

While dementia science recognizes the existence of multiple dementias, each seems to press the case for a “predictive” biomedicine, characterized by discourses of prevention, risk and uncertainty (Gottweis 2005). While the continuum hypothesis, for example, helps trouble the premise that ADAD is just a natural consequence of getting old, thereby troubling the ageism inherent to clinical medicine (Oliver 2008), the hypothesis also gets scaled up in “apocalyptic” (Robertson 1991) predictions over population ageing, and the costs of Alzheimer's disease (AD) and dementia (see e.g. Alzheimer’s Disease International 2015). Specifically, an emergent “actor-network” of dementia science, at the interface with biomedicine and public health (Latimer 2018) constructs dementia as multiple, while at the same time helps create a strategy of early detection and predictive technologies that can identify people at risk of ADAD *before* they become symptomatic (Bloom 2014; Milne 2016; Moreira, May, and Bond 2009; Swallow 2016).

To meet the pressure for early detection strategies there has been an emphasis on research that can identify bioclinical entities that cross between the clinic and the laboratory, such as mild cognitive impairment (MCI) (Moreira, May, and Bond 2009). Bioclinical entities are hybrid platforms through which the laboratory and the clinic are bridged to help stabilize a diagnostic category. Moreira *et al.*’s model of a bioclinical entity such as MCI stresses the intersection of the laboratory, the clinic, industry and mechanisms of regulation in networks of interdependence. But in the context of early detection of dementia, as discussed above, there is increasing pressure for bioclinical entities that can be used to identify those at risk of dementia *before* they become even mildly clinically symptomatic. Specifically, the emphasis is on establishing biomarkers of dementia as bioclinical entities that can help stabilize *preclinical* dementia.

Leibing (2016) suggests that biomarkers of dementia have changed little between 1993 and 2013. She identifies the main diagnostic biomarkers associated with dementia as genes (such as ApoE4), Amyloid and Tau proteins (present in, for example, cerebrospinal fluid, as well as plaques and tangles in the brain) and brain atrophy, visible on the new generation of MRI and PET scans (Leibing 2016, 46). Leibing suggests that these biomarkers are there to support diagnosis, and that they have failed to reach enough strength or specificity to be definitive on their own. However, these biomarkers, as Boenink (2016) suggests, to have predictive value (however stochastic or probabilistic) need to have their associations with clinical symptoms validated. Moreover, because biomarkers seem to create further opportunities for uncertainty and the need for interpretation over the difference between susceptibility and risk (see also Lock and Hedgecoe 2009) questions arise as to how they are to be made stable *enough* in the diagnosis of preclinical dementia. Which brings us back to the debate over how biomarkers are being validated for use in the early detection of dementia somaticize dementia.

In addition to examining how biomarkers of dementia are used in the laboratory in research using animal models, we also examine how biomarkers are used in the clinic. In what follows, we explore the different ways in which the association between biomarkers and dementia is deployed in the laboratory and in the clinic. Specifically, in respect of the taken for granted need to stabilize the association, we explore what is included and what is excluded for the association to hold. Hence we do not assume these inclusions and exclusions to simply represent “short-cuts” to irresponsible innovation (Leibing 2016). Rather, our concern in this paper is to explore why genetic biomarkers alone may not be stable enough to, as Leibing puts it, reach enough strength or specificity to be definitive in the early detection of preclinical dementia in humans.

## The studies

In the paper, we juxtapose our ethnography of memory clinics, funded by The Wellcome Trust, with our research on biology and ageing funded by the ESRC and Latimer’s current institution.

Fieldwork undertaken by Hillman in memory clinics based in two large Regional National Health Service hospitals attached to Medical Schools in the UK, was carried out between 2012 and 2014. While memory clinics were well established as part of secondary medical services specializing in older people for some time, they have expanded in line with pressure from governments and public health policy for early detection technologies, discussed above, with the aim of diagnosing people *at risk* of developing dementia. Our two clinics are both representative of hospital-based memory services across the UK and each functioned in similar ways, namely assessing patients experiencing problems with thinking and memory. The fieldwork involved the audio recording and the taking of ethnographic fieldnotes of clinical consultations (*N* = 51) and interviews with 13 memory clinic staff, 21 patients, 19 relatives/carers. The directors of the 2 memory clinic research sites were also active in dementia science, and identified as having international influence in the field. Additionally, Hillman identified 10 further dementia scientists from the research and clinical practice networks of the 2 directors, all UK-based, for participation in interviews. Their areas of expertise included: public health, genetics, psychiatric genetics, base biology, clinical trials and a combination of these. Half of those in the sample were also clinically active in dementia diagnosis and treatment alongside their research practice. They were all, like the two directors, part of international collaborative networks in Europe and worldwide, including large studies that brought different specialisms together. Henceforth we refer to those participants in our study that were active clinically and as scientists “clinician-scientists.”

Alongside the memory clinic study, we draw on the analysis of animal models of ADAD used in two laboratory experiments. Since 1995 transgenic mice and other animal models of different forms of ADAD are a common feature of basic research in neurobiology, particularly in relation to the identification of those genetic mutations associated with different forms of dementia. More recently there has been pressure for the models to be developed that can help identify biomarkers of presymptomatic dementia (King 2018). Our examples draw on Latimer’s extended ethnography of biology and ageing (2009–2017) (Latimer 2018, 2019), which has included site visits, interviews and extended conversations with over 30 scientists across the UK and US investigating the biology of ageing and associated diseases, as well as analysis of publicly available representations of animal model research on ageing, and most recently two years of participant observation of scientists working on ageing at a prestigious UK Life Sciences Institute.

For both ethnographies, analysis began in the field where interpretations were shaped and informed by ongoing relationships. Memory clinic consultations were audio recorded and transcribed, fieldnotes from all sites were written up immediately following periods of observation. These texts were collated and analyzed for the various ways in which ADAD were being enacted, in order to identify key themes. These were compared both within and across the different field sites. In the memory clinic study, key themes were reflected in the assessment and diagnosis of those who were in the early stages or at risk of dementia *as well as* those with established dementia. In the laboratory-based study, how etiologies of neurodegeneration and ageing were being researched and accounted for were identified and analyzed for the different ways in which they use biomarkers and enact dementia, as, for example, universal, genetically determined effects, or as situated, localized and specific. We have chosen extracts from our fieldwork that represent identified key themes from the wider body of ethnographic material.

## Making dementia hold in the clinic

Biomarkers are enacted in the clinical context as “provisional” rather than definitive (Biomarkers Definitions Working Group 2001), with their significance needing to be grounded. Indeed, in the memory clinics we studied, genetic biomarkers such as those described by Leibing above, are not used in the diagnostic process, although the same clinician–scientists as we saw working in the clinic may use them in their laboratory-based research. Rather, our clinician–scientists made stark distinctions between the role of biomarkers in research, for identifying those at risk of future dementia, and the drive to firm up the connections between biomarkers and symptomatic expression of diseases causing dementia, and their reliability to give credence and a greater degree of certainty to a diagnostic label in the clinic. For example,

Alex Hillman (AH): [what] I haven’t managed to get my head around is the changes that you can see, like you were saying about amyloid changes through imaging and things. But how that relates to symptoms experienced and … 

Clinician-scientist: Amyloid doesn’t particularly at all. So, we know from fixed studies that you can have a lot of amyloid in the brain but you can be completely cognitively normal. So, it’s just not specific enough. I mean the brain imaging is an interesting one because again, I mean I always go on the clinical picture rather than a brain image. So, I’ve had - again you get second opinions. I’ve been asked to see somebody who the clinician wants to say they’ve got dementia based on their scan. The scan is showing the brain atrophy which is compatible with a diagnosis of Alzheimer’s. But then when you look at and talk to the patient and you examine the patient technically, and do cognitive tests they are brighter than you are. They are absolutely totally switched on. There is nothing wrong in any way or form you could pick up …  … So people, that’s why I’m a little bit negative about any biomarkers because ultimately even on land, I mean if you look at that scan in isolation you can totally see why the clinician would say, that person looks to have dementia. They haven’t. They haven’t and that’s the bottom line.

In this extract, the clinician–scientist warns against the utilization of biomarkers in isolation, biomarkers which can stand in direct contradiction to the “real world” patient they see in front of them. In medicine, and particularly in the context of ADAD, clinical judgement, and the reading of the patient, takes precedence over individual tests, scans or biomarkers. If the patient is not “dementing,” they don’t have dementia “and that’s the bottom line.”

This “bottom line” however sits uncomfortably alongside a research agenda in which risk profiling for dementia is being championed through the firming up of biomarkers as having some predictive value. That is not to say that the biological is entirely absent in the context of clinical diagnosis of dementia. It is rather that making associations between the biological and the symptomatic is complex. The biomarkers of interest in the clinical context involve Computerized Tomography (CT) scanning of the brain, which are read to identify any atrophy, and the areas of the brain which may be affected by it, and blood tests, mainly undertaken as a means of excluding other potential causes of a patient’s presenting problem. Alongside these diagnostic tests, clinicians take a detailed patient history from patients themselves and often from their relative or carer who has been asked to attend with them.

The patient history is interpreted together with cognitive tests, commonly undertaken in a separate adjoining room by a psychologist or nurse practitioner but sometimes carried out by the doctor or psychiatrist as part of the consultation. The cognitive tests are designed to test particular aspects of thinking associated with dementia including memory recall, perception and orientation and word finding/aphasia which are often made to connect to different parts of the brain – something that can later be fitted to the CT scan of the brain. As the extract above makes clear, the biological markers are of little significance in and of themselves, without the patient history and cognitive tests to give them form and meaning.

The clinical context, therefore, requires a substantial degree of interpretive work to bring together and assign significance to the various pieces of the puzzle that help to articulate dementia diagnostically. However, clinicians also engage patients and their carers in extensive interactions over the meanings and significance of these histories and of the tests (Hillman and Latimer 2019). Indeed, patients are brought into play in interactions in the clinic as subjects who are situated in biography and milieu, but also as having the capacity to reason, think, feel, will, perceive, judge, sense and make sense. As one clinician explains, even the cognitive tests are limited in their capacity to steer a diagnosis, without their relationship to good clinical history, often including a “collateral history,” of what is happening in a patient’s life being established:

Well I think the main sort of point to start with assessing people with dementia is definitely to get a history rather than go straight for a memory test. So, a history that you can have in part, partly from the patient but it’s very important to get some sort of collateral history from a person who knows the patient very well. So, normally yes, we ask when we send the referral, the appointment letter you know if it’s possible to come with a person, a carer, a spouse, or a friend or whoever, that knows you well, to be able to give a collateral history because of the nature of the condition. Some patients even are in complete denial of having any problems. So that’s probably the most important part of the assessment is to have a good history of events. And then you go to do some proper cognitive assessment which mainly sort of normally reflects what is happening in real life. (Memory Clinic Consultant)

Here the clinician describes what they see as the key aspect of building a dementia diagnosis, assigning the cognitive assessments and other kinds of tests and markers as secondary to the gathering of good history.

The tests become useful as a confirmation of “real life” as it is presented through the course of clinic interactions. The clinician speaks of the patient as a person who can give a history but also as someone whose history needs to be confirmed by someone else – both patient and family member are brought into play as thinking, knowing subjects – people who can be “known well,” or who can be “in denial.” Cognitive scores are described as the best mechanism through which to connect the biological to the experiential. On the one hand, aspects of the thinking that are shown to be impaired through the test scores can be made to connect to the brain scan – showing the parts of the brain likely to be affected and thus producing the particular impairment evidenced through the test. On the other hand, the test can be connected to the experiences that patients and families describe, such as not being able to find the right words or the names of things or people. However, the cognitive score, as with the brain scan, can also be treated provisionally in the memory clinic as a tool requiring interpretation according to the “reading” (Atkinson 1995) of the individual patient and their family as minded and as situated:

Some people in earlier stages that clearly have the deterioration in cognitive function is starting to have some impact on everyday functioning. And you will say, sort of say that “yes, definitely sort of early stages of dementia.” Sometimes it doesn’t translate on the formal cognitive assessment and people do very well. People with a good level of education and ability can score within normal limits you know for some time. So that’s why the history is so important*.* (Memory Clinic Consultant)

Here, social concerns including the level of education, are made central to practices of clinical judgement that are drawn upon to mediate and nuance interpretation of formal tests, including the cognitive test score. Like much medical sociology has shown, clinical tests are mechanisms through which to ascribe significance to or challenge patient disposal (Berg 1992). This is particularly prescient in the case of ADAD, where the tests and biomarkers of disease carry so much uncertainty and thus require a greater degree of interpretative work to connect them to the presentation of the symptomatic expressions of illness (Whitehouse, Frisoni, and Post 2004).

We saw across all our observations of interactions in the clinic that patients and their family are indeed engaged in creating a picture of the patient – they are engaged as subjects with the ability to know, make sense, represent. Critically, interpretation of biological markers, as well as cognitive scores, are *situated*, in and by the making of histories through interactions. Thus, through the taking of histories and the interactions in the clinic, the patient and their family are engaged as minded subjects, who can recall, reflect, understand, feel. Diagnosis is thus best understood as an “event” through which dementia is or is not made stable: an event that brings us closer to calls for locating dementia as pertaining to minded, situated and embodied subjects.

## Dementia, animal models and hypothetical pathways

As already discussed, there is increasing interest in identifying biomarkers that can signify dementia prior to the development of any symptoms, to enable, as this clinician-scientist puts it, early intervention:

I mean it’s all based on the hypothesis that actually, and it may not be true, but it may be too late once the disease is well advanced. And our only hope really is around very early intervention. That’s where the smart money seems to be moving, towards very early intervention. (Clinician-scientist)

However, the logic here around persons having dementia runs conversely to that which was advocated by clinicians in the previous section. Rather, the aim, as another clinician-scientist put it to us, is for the people taking part in his research to be defined as having AD even though they are symptomless:

People in the project will have Alzheimer’s Disease- by definition- they’ll be biomarker positive, they’ll have evidence of amyloid and tau on their brain but they don’t have dementia. We’re trying to prevent dementia.

The aim of contemporary research according to this clinician-scientist is that humans need to display evidence of dementia as biomarkers, and even display some morphological changes to, for example, the brain, but they do not need to display any clinical symptoms in order to “have” the disease. Doing this in humans is problematic because, to quote another participant, longitudinal studies in humans that firm up the associations between the biomarkers and development of symptoms “run for decades” and there is a need now for drugs that can effect AD *before* a person becomes cognitively impaired.^1^

Laboratory experiments using animal models of ADAD are being sought that can help to firm-up the association between genetic biomarkers and identifiable symptoms (King 2018) that do not “run for decades.” For example, transgenic mouse models are used across dementia science research (Perry, Zhu, and Smith 2013), one of which was developed in a molecular biology laboratory at the Max Plank Institute in Hamburg.^2^ The mouse’s genes have been made to “replicate” AD by being genetically modified to “have” the human tau gene, and experiments that result in defective phosphorylation of tau and accumulation of neurofibrillatory tangles. This model is aimed at enabling researchers to investigate cognition and behavior (aspects of the phenotype) in relation to the genotype and its expression in the mice. Controls – the “normal” wild type mice – systematically bury marbles in their bedding. In contrast, the transgenic AD mice are disorganized and unsystematic – they cannot bury marbles in an ordered way but just rummage around in their bedding. This inability to bury marbles is taken as evidence of loss of cognitive function: in other words the experiment associates “symptoms” in mice with the presence of the genetic biomarkers of AD (the defective human tau gene). The scientists succeeded in demonstrating that once the defective tau-gene is deactivated, transgenic mice, which previously presented symptoms of dementia, regain their cognitive abilities. This raises the possibility for the scientists of whether medicines can be developed which similarly can both deactivate the defective Human tau gene and do so prior to irreversible cognitive damage.

In this example, cognition is constructed as a function of genetic material that activates/deactivates biological processes in the brain and which in turn are associated with symptoms of dementia. This requires the blackboxing of long chains of translation (Latour 1987): such as those connecting AD- type dementia with tau proteins through experiments with transgenic mice modified to “have” the human tau gene; and the “effort,” as Rheinberger (2010) calls it, that goes into the experimental system that it helps to generate (Davies 2012). Through this blackboxing, biomarkers of AD (the defective human tau gene) can come into view, for a moment at least, as stable entities, and even eventually as predictive of the risk of dementia. Notably, the cognitive dysfunction that tau produces in mice is observed through changes in their behavior – that the transgenic mice who have the human tau gene are unable to systematically bury marbles as they would “normally” is for all intents and purposes a symptom. Here then the mouse as a surrogate patient (Lewis *et al.* 2013) helps firm up the association between the biomarkers and clinical symptoms of cognitive decline. What is enacted is a neurocognitive mechanism that produces a relation between genotype and phenotype, but what is made absent by this enactment is a situated, minded subject: the mouse is not attributed with the capacity to think, nor are they situated as a social, embodied subject, indeed the social life of the mouse is of no interest to the experiment.

In the Max Plank Institute experiment, making dementia hold as an association between genetic biomarkers and symptoms appears relatively straightforward. Aspects of organic material in the mice’s brain is made to represent dementia and the symptoms of neurological dysfunction (the inability to bury marbles in an organized way) provide the evidence for the effects of the defective Human Tau gene. The lifespan of the mouse (2 years maximum) truncates the need to trace the relationship between biomarkers and the progression of dementia over time. A degree of simplicity that is severely lacking in human subjects. What is also made absent is any sense of the mice as either minded or situated subjects – on the contrary, it is the defective genes which are attributed with the material agency to affect cognition and behavior, although the pathology can be reversed through reengineering the mouse’s genetic makeup.

## Gene expression, plasticity and variation

Next, we present research in the biosciences of ageing which focusses on epigenetic biological processes, and which associates particular genes with signs and symptoms of neurodegeneration in nematode worms. For bioscientists interested in ageing, neurodegeneration may be one of many effects of the underpinning biological processes that cause organisms to age well or badly. Questions arise regarding how the specific effects (such as signs of neurodegeneration), visible at the level of the phenotype, are produced.

In the following extract a visiting molecular biologist, Mary, is presenting and talking about her work on neurodegeneration and ADAD diseases with members of the host laboratory (a small group of six people), which is part of a larger epigenetics group and that constitutes a “worm lab.” The nematode worm, *c. elegans*, is the animal model that connects the work of the host and the visitor, together with an interest in ageing and neurodegenerative processes. The nematode worm is an experimental model in molecular biology because it shares some basic biological mechanisms with many other species including the human (an evolutionary effect known as “conservation”). In traveling from the mice to the worm, we are traveling back in evolutionary time and while this might seem reductionist it also means that what can be opened up for scrutiny are some of the seeming “short-cuts” (Leibing 2016) being blackboxed in the reported experiments with mice. Specifically, in what follows, what comes into view is how genes do or do not get expressed (penetrate the phenotype) over the lifespan of the animal and what the effects of these expressions mean in terms of the phenotype and its neurobiological behavior.

Mary tells us that her research explores neurodegeneration and toxicity associated with TDP–43 (a protein that in humans is encoded by the TARDBP gene). She says that TDP-43 effects different kinds of neurones and is associated with different motor-neurone functions. In “diseased bodies” TDP-43 aggregates – there is an absence of RNA that stabilizes and stops the TDP-43 from aggregating and forming clumps. She says that she has been working with a wild type (WT) of nematode and a generated strain of TDP-43 expressing nematodes. This latter worm is a homolog for human TDP-43. She says that TDP-43 worms lay 50% less eggs, and only 60% of their eggs hatch (as opposed to 100% in the WT), and that TDP-43 expression also affects ageing and lifespan with a decrease in median lifespan. She says that she has been able to show that neurofunction is also affected by TDP-43 expression. Specifically, motor function, measured by counting the number of thrashes when the worm is stimulated, is affected by TDP-43 expression: specifically, TDP-43 expressing worms thrash less than the wild-type worms, and she shows a film of worms thrashing at different rates. She states that they also tested the worms crawling speed – she says you can check their swimming speed but crawling speed is easier – the crawling speed is slower in the TDP-43 expressing worms.

In the second experiment, the phenotypes between Wild Type and TDP-43 worms were synchronized at day 1 of their lifespan. This time Mary’s lab checked for morphological differences (visual changes to the worms’ neurone) first – she reports that they did not see any differences. Then they tried pushing the worms age to 5 days – so that they were looking at “aged worms” – to see if there were any morphological differences in degeneration or death of neurones, and they found that there were. They then checked mechanistic neurofunction (as above) and this was reduced in TDP-43 expressing animals. Then they asked: “are neurones defective in chemosensory functions?” The test used repellent odorants and showed that avoidance response is reduced in TDP-43 expressing animals – indicating that they had become less sensitive. In a rare moment when the animal is attributed with some sense of mindedness Janice, the head of the host laboratory, asks if they might be “clever” worms – “those worms who have loss of sensory function might live longer?” Mary responds saying that on the contrary loss of olfactory function is linked to premature ageing and even death.

These experiments – like many being undertaken across the globe – are able to track the relation between specific biomarkers – in this case, TDP-43 – neurodegeneration and the temporalities of complex biological processes that underpin these associations. What these biologists are enacting is a way of opening up the complexity of how genes work, particularly with respect to the protective mechanisms involved in how genes and gene mutations control proteins including their aggregation. Aggregation of proteins forms clumps, clumps are enacted as what kills neurons to form atrophy. Specifically, in the comparison between transgenic and wild type worms, as well as between worms of different ages, Mary shows how mutations in the TARDBP gene coupled with changes over time (age) affect transcription and how proteins are regulated and as a result of which the neurocognitive function of the worms is disrupted. But what she is also enacting is plasticity and variability over gene expression.

The problem for biomedicine is how to mobilize the experimental model of the genetics of ADAD as a “spokesperson” (Latour 1987) for dementia in *humans*. On the one hand, there are problems over making the crossing between brain and mind (Williams, Katz, and Martin 2011, 2012). It is this crossing that holds particular challenges for modeling neurodegeneration, with many mice models showing promise for treatment strategies that have proved to be ineffective in humans resulting in a “repository for frustrated hopes” (Milne 2016, 397). Critically, Mary makes the crossing back to the human, not through reintroducing the minded subject but by reminding her audience that the gene TDP-43 is associated with frontal-temporal lobe dementia (FTD) in humans, that humans identified as having frontal–temporal lobe dementia have been shown to express TDP-43 (see also Ling *et al.* 2015). Mary thus enacts her research as opening up associations between genes, mutations and how protective biological mechanisms work or do not – and it is the protective mechanism at the biological level that seems to fail in humans too in the production of the neurodegenerative condition called FTD. This work overlaps with Johnson *et al.*’s (cited in Leibing 2016, 53) longitudinal study of the genome of 2000 human individuals that showed that some individuals have a genetic mutation that protects them from the amyloid build up with none of those with this protective mutation suffering from AD, or cognitive decline. The difference is, Mary’s work only takes a matter of weeks not “decades” to achieve. But neither Johnson nor Mary are claiming that the genetic biomarkers involved can simply be used as predictors of AD or FTD in humans. On the contrary, Mary is putting together a complex set of variables, including age, to suggest that as the animal ages there are changes in the protective mechanisms that prevent protein aggregation in worms that express TDP-43.

## Post field-work reflections

Before ending it is important to reflect further on the animal modeling that underpins laboratory attempts to stabilize biomarkers of different forms of ADAD that can be used in early detection, and the paradigms that inform clinical diagnosis. After setting the scene for why genetic biomarkers have become so important to dementia science, we then discussed the disjuncture, as noted in the literature, between the paradigms that inform clinical diagnosis and the animal modeling that underpins laboratory attempts to stabilize genetic biomarkers of ADAD for early detection. Specifically, pressure for early detection technologies has been critiqued by medical doctors as well as social scientists for their reduction of dementia to biological mechanisms in ways that do not account for how minded and situated personhood unfolds over time.

Following the section that introduced our three research sites, we first presented an analysis of clinical discourse and practice in memory clinics dedicated to the early detection of dementia. Here we showed how genetic biomarkers of the problems patients are experiencing with memory and thinking are made absent. Instead, for a diagnosis of dementia to hold it is the association between the patient as a minded, social subject, situated in biography and milieu, and somatic evidence of dementia located in pathological changes to the brain, that stabilizes a diagnosis of dementia. We then went on to illustrate how dementia science in pursuit of possible routes to therapeutic interventions tests tau’s association with AD, the most common form of dementia, by reinforcing the association of genetic biomarkers with symptoms of dementia in mice. This process does not attribute mice as either minded or situated so by default elides the minded, situated subject upon which the clinic relies to firm up a diagnosis of dementia. Next, we presented new research in the biosciences of ageing which focusses on epigenetic biological processes, and which associates particular genetic processes with symptoms of neurodegeneration in nematode worms. This research presses the relationship between gene expression and age (early and later in life), and makes some attempt to situate the biology of dementia, but does not attribute worms with any form of mindedness.

Both these laboratory studies attribute no mindedness to the animals they use, rather neurological symptoms are conceived of as “behavior” and attributed to what is hardwired cognition. This, of course, raises the problem of how to make an animal or any other kind of model an analog for human mindedness, let alone the inter-relational complexity of the mind, memory, and illness upon which meaningful clinical work with people with neurological disease seem to depend. However, in our shift to basic molecular neuroscience research what we showed is that the laboratory-based enactments do not align with the straightforward picture of biomarkers as predictors of dementia – the picture that is being optimistically perpetuated in policy discourse.

While the same biomarkers are not necessarily in play in each domain, what emerges in our study is how the clinic refigures some of the “messiness” of minded, located personhood that the laboratory cuts out, as at the same time as it makes absent the messiness and uncertainty of the genomic underpinnings of dementia that the laboratory includes. However, with the rise of epigenetics, it seems that some of the “chaos of life” (De Beer 2013, 2) has been brought back into the laboratory. In particular, scientists embed molecular processes of neurodegeneration in the specificities of the animals’ “biography” and “milieu” (Niewöhner 2011), not as minded subjects, but in relation to gene-environment interactions over time.

In Mary’s research, as well as in the Max Plank work, genotype-phenotype relations are being enacted as variable and plastic: AD can be reversed in worms once the Human Tau-gene is neutralized, while TDP-43 expression changes with age in nematodes. Questions arise as to whether research on the molecular basis of ADADs disrupt understandings of neurodegeneration as necessarily having a continuous, irreversible and inevitable trajectory, or even that ADAD are the effects of mechanisms internal to an organism’s brain. In the research being undertaken in the host Life Sciences institute in which Mary is giving her paper, what is being focussed upon are variability and plasticity in how gene expression is affected by the environment in which the animal is situated (their milieu) across the animal’s lifespan. For example, experiments were being conducted in which the animals’ environments (in the form of food, heat and so on) were modified and which explored the effects of this on the kind of molecular processes described by Mary above. So not only one version of their “biography” (their genetic origins and their life over time) but their milieu (their environment) entered into the deliberations over the correlations between neurological effects and gene expression.

Mary’s research is concerned with how the materialities of the world (bodies, genes, environmental forces) interact and are co-constitutive of biological processes – for example in the relationships between neurodegeneration, gene expression and age. Does this shift represent the possibility that dementia could move towards more situated biology? A shift that was hinted at in an interview with another molecular biologist:

The human population is not under lab conditions, there is incredible variation in their environmental exposures to things. There is a lot of genetic variation to begin with and I think that they are coming against a degree of complexity that is really really tough for them (bioscientists) to nail down and say “yes, this gene here is a determinant of longevity in human populations.” I mean some of it has been published but I think that they all feel a level of discomfort that they haven’t found big effects like we find in worms and flies and I think the complexity of the environment, so this gets back to you know, when we go “Ah ha there are genes that determine how long animals live,” and we talk, we use this for determine, determine, determine, determine. That is all very well but we have got to take a step back and say “Yes it determines it in that very specific environment in the laboratory. Now if you broaden this thing out to a number of different environments, diet, nutrition, stress, blah, blah, does it still hold true that this gene determines lifespan?” I think that the answer is “No.” Different genes under different circumstances, very complex picture of genes interacting with environmental factors producing an ageing outcome. And it seems like everywhere we look in the lab for things that could modulate ageing, so you know early exposure to radiation, early exposure to stress, we see changes in lifespan all the time and it makes you wonder why people weren’t working on this a 100 years ago. (US based biologist of ageing)

In this account, the biologist is pressing how biological processes affecting how organisms age is relational, plastic and variable. It seems as if he is suggesting that some of the “chaos of life” needs to be allowed to enter the laboratory. But he, like Mary and our host Life Sciences Institute, conceives of the environment almost entirely in terms of “natural” forces (Niewöhner 2011): the laboratory animal has a biography of sorts (a genetically engineered biography perhaps) and a milieu (as environment made up of natural forces), but unlike in the clinic the animals being modeled are not enacted as minded subjects.

The shift towards more situated and temporal understandings of the processes underpinning effects such as neurodegeneration resonates with a growing body of literature that seeks to understand the implications of epigenetics as a more relational approach to genomics and genetic expression for understanding long-term health and illness processes such as dementia (e.g. Müller and Samarasa 2018; Niewohner 2015; Palsson 2016; Pickersgill *et al.* 2013). In particular, there is interest in whether the genome is being understood as highly sensitive “to social influence,” including how “social regulation of gene expression is a potent influence on behavior” (Robinson, Grozinger, and Whitfield 2005, 258). This shift towards situated biology, or even perhaps eventually a “biosocial genome” (Müller *et al.* 2017) makes attempts to stabilize genetic biomarkers of preclinical ADAD even more problematic. Specifically, where the laboratory science seems to be moving towards conceiving of biological processes that underpin particular pathologies as *located* in gene-environment interactions across the lifespan of the animal, including possibilities for variation, plasticity and even reversibility of neurodegenerative effects, do genetic biomarkers as is the case in the clinic, need to be treated as always provisional?

## Concluding thoughts

Both inside and outside the laboratory, in biomedical and public health research related to dementia, much is being invested in the hypothesis of pre-clinical dementia and the establishment of biomarkers of a disease that takes years to develop into a symptomatic illness. The pursuit of biomarkers that can indicate the presence of neurodegenerative disease years ahead of someone’s experiencing any symptoms of being ill, accords with contemporary forms of anticipatory (Adams, Murphy, and Clarke 2009) or pre-emptive (Massumi 2007) biopolitics. But for our participants in clinical work, as well as in the science at the interface with the clinic, the associations between these biomarkers and the symptoms of ADAD in humans must also at moments be made to “hold.”

One of our aims has been to explore the disjuncture between the animal modeling that underpins laboratory attempts to stabilize genetic biomarkers of ADAD for early detection and the paradigms that inform clinical diagnosis. Specifically, we discussed how pressure for early detection technologies are critiqued by medical doctors as well as social scientists for their reduction of dementia to biological mechanisms in ways that do not account for how minded and situated personhood unfolds over time. Our second aim has been, drawing on Niewöhner and Lock’s recent work (2018), to explore the extent to which dementia is also enacted as relational, the effect of gene-environment interactions that unfold over time, thus getting us beyond not just the constraints of genetic determinism, but also beyond the constraints of too individuated a notion of mindedness as a biosocial effect.

In this respect, we showed how the diagnostic process that we observed in the clinic entails interactional work that associates, or not, diverse biomarkers with patients’ and families’ accounts, in ways that bring us closer to an idea of a minded dementia subject. This process does not use genetic biomarkers. Dementia is still located, however, in an individual brain, but that brain is enacted as belonging to a *specific* and minded human subject. In the AD laboratory, we showed how dementia is enacted as the effect of specific defective genes, associated with symptoms in mice – namely failures of behavior and cognitive function. Mice were not however enacted as in anyway minded – “behavior” was being performed as genetically determined and mechanistic. So the laboratory includes what the clinic cuts out, while the clinic includes what the laboratory cuts out.

It is unsurprising that the translation of biomarkers varies across the clinic and the laboratory. While the general uncertainty associated with biomarkers has been noted (e.g. Metzler 2010), it is crystallised in dementia science in which diagnosis is complex, and the relationship between the biological and mindedness “entangled” (Lock 2014). In the context of dementia laboratory research biomarkers are problematic because of the problem of calibrating animal and human disease (Milne 2019), while in the clinic biomarkers are contextualized and their meaning contingent (see also Swallow 2019). Moreover, biomarkers in ADAD, as this special issue explores, are doubly problematic because it is not clear even what they are markers of, and how the processes that they mark relate to the condition, its etiology and its unfolding. While existing attempts to “biomarkerize” (Metzler 2010) preclinical dementia are ongoing, their stabilization seems to us to be failing from the perspective of clinical practice, which needs to situate bodies and minds. It would also be useful for future research to consider what the existence of symptomless patients who test positive for biomarkers mean for biomarker development.

We went on to show how in our third site of enactment, an epigenetics “worm” laboratory concerned with the biology of ageing, neurodegenerative changes are located in gene-environment interactions over time, and discussed how a shift to epigenetics enacts the environment or “milieu,” as “natural” forces. We reflected on whether such an enactment could bring us closer to a notion of “situated biology” and possibilities for the biology of dementia to be understood as relational. This would also offer possibilities for biology to come a little closer to understandings of mindedness and memory as also relational and situated (see e.g. Brown and Reavey 2015).

We want to make an albeit speculative suggestion: working with animal models to identify and understand the underpinning biology – genes, their expression and how they regulate biological processes, such as aggregation – involved in producing the conditions underpinning neurodegeneration may make possible examination of the more situated and relational aspects of these processes. Rather than firming up the grounds upon which biomarkers can become predictive of different kinds of ADAD, animal model experiments in epigenetics seem to articulate gene expression and its disruptive neurological effects in complex ways. Further social science research could explore whether epigenetic experiments with animal models firm up the *specificity* of biological processes underpinning neurodegeneration as situated, plastic and variable. Of course, the minded subject is made absent in the laboratory but what is sometimes made present is that gene expression is not pre-determined, and that the genotype-phenotype relation in ADAD is also relational, temporal and plastic.

## Disclosure statement

No potential conflict of interest was reported by the authors.

## Ethical approval

This project successfully gained ethical approval from the NHS to carry out the work, including informed signed consent from all participants. All aspects of this research successfully gained ethical approval from two University ethics committees, and informed consent from all participants.

## Notes

1 In our study of memory clinics, the clinician–scientists that we interviewed were mainly involved in longitudinal studies aimed at identifying risk factors for dementia, including associated genetic biomarkers, such as those taking place at ULC’s Dementia Research Centre (https://www.ucl.ac.uk/drc/research-studies).

2 Max Plant Research Unit for Structural Molecular Biology at DESY in Hamburg, where scientists are now testing active substances to prevent the formation of tau deposits in mice. https://www.mpg.de/1161288/alzheimer_reversible_memory_loss.
